# Supplementary material for: Estimating the impact of influenza on the epidemiological dynamics of SARS-CoV-2
Source: PeerJ. 2021 Dec 3;9:e12566. doi: 10.7717/peerj.12566 (PMC8647717; doi:10.7717/peerj.12566)
Supplement: Supplemental Information 1 [file peerj-09-12566-s001.pdf]

Estimating the impact of influenza on the epidemiological dynamics of SARS-CoV-2

Supplementary Materials

Authors Matthieu Domenech de Cellès[1], Jean-Sébastien Casalegno[2,3], Bruno Lina[2,3], Lulla Opatowski[4,5]

Affiliations

1. Max Planck Institute for Infection Biology, Infectious Disease Epidemiology group, Charitéplatz 1, Campus Charité Mitte, 10117 Berlin, Germany
2. Laboratoire de Virologie des HCL, IAI, CNR des virus à transmission respiratoire (dont la grippe) Hôpital de la Croix-Rousse F-69317 Lyon cedex 04, France
3. Virpath, Centre International de Recherche en Infectiologie (CIRI), Université de Lyon Inserm U1111, CNRS UMR 5308, ENS de Lyon, UCBL F-69372 Lyon cedex 08, France
4. Université Paris-Saclay, UVSQ, Univ. Paris-Sud, Inserm, CESP, Anti-infective evasion and pharmacoepidemiology team, F-78180 Montigny-Le-Bretonneux, France
5. Institut Pasteur, Epidemiology and Modelling of Evasion to Antibiotics F-75015 Paris, France

S1 Supplementary data and methods

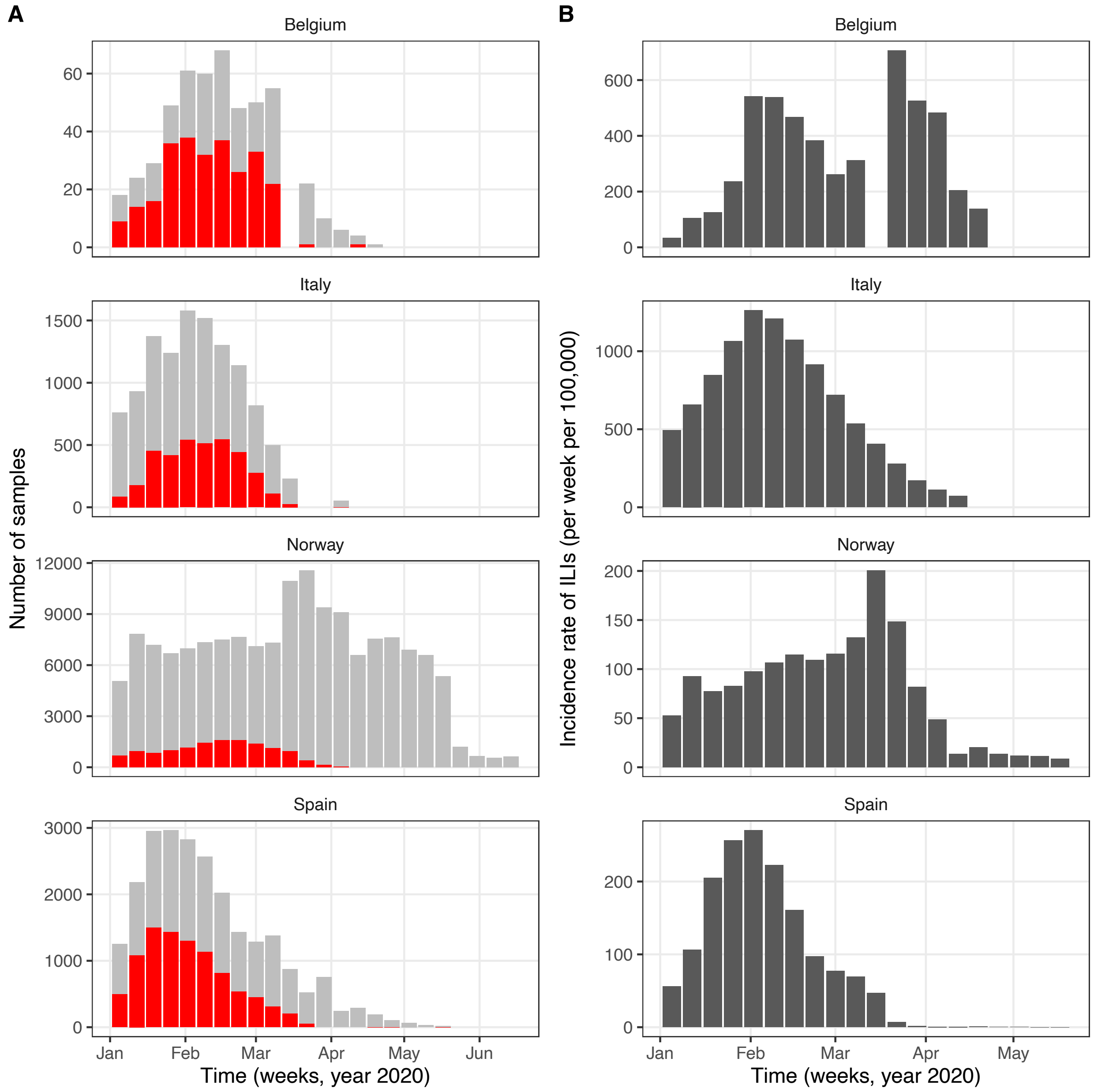

Figure S1: **Syndromic ILI data (A) and virological influenza data (B) in Belgium, Italy, Norway, and Spain**. In A, the red bars represent the numbers of samples positive to any influenza virus and the grey bars those negative.

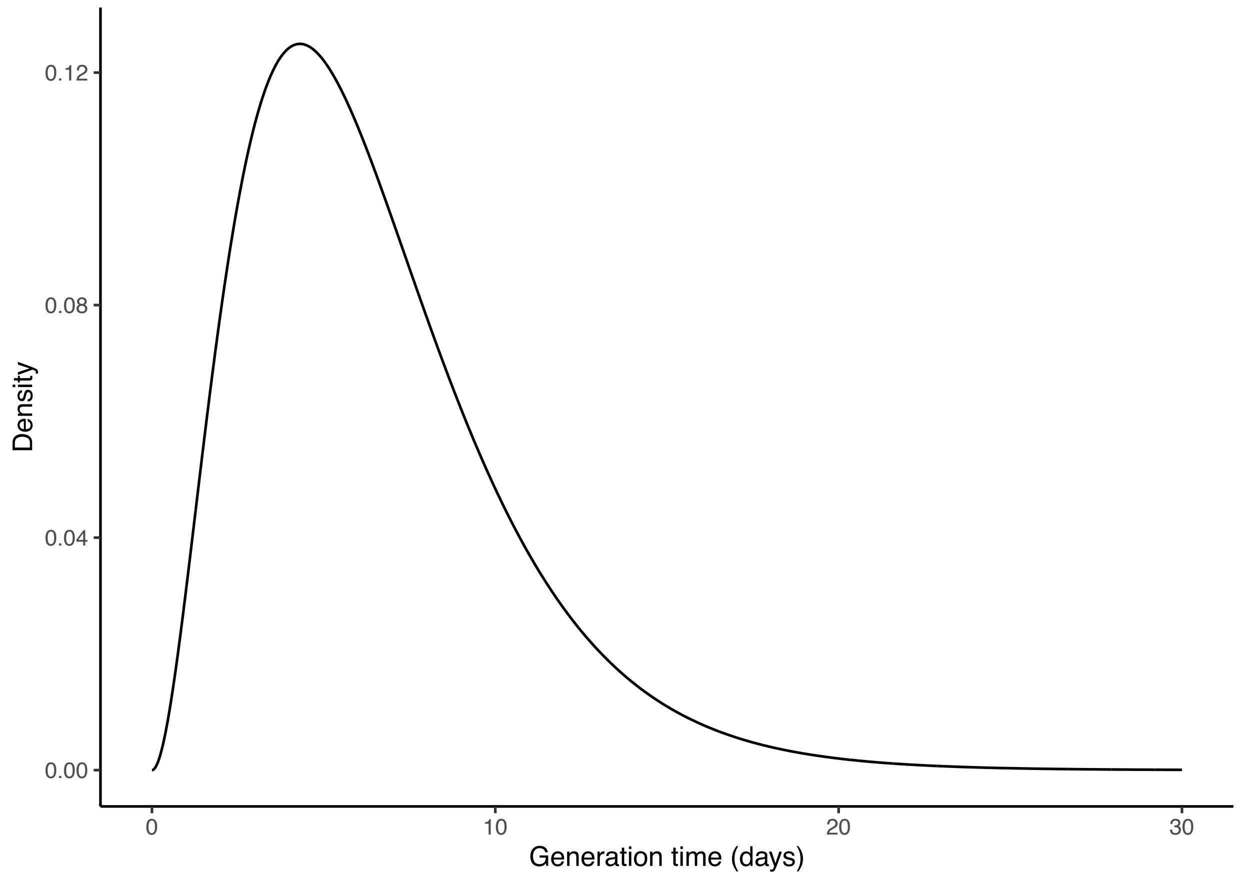

Figure S2: **Model-based distribution of the generation time**. For our model, the density function of the generation time is given by [Camacho *et al.* , 2011, Svensson, 2007]: $f(t) = \frac{4\sigma^2\gamma}{(\gamma-\sigma)}[te^{-2\sigma t} + \frac{1}{2(\gamma-\sigma)}(e^{-2\gamma t} - e^{-2\sigma t})]$. The resulting distribution is bell-shaped, with mean $\sigma^{-1} + \frac{\gamma^{-1}}{2} =$ 6.5 days and coefficient of variation 0.58.

S2 Supplementary results

Log-likelihood profiles of β_F The log-likelihood profiles for the impact of influenza (parameter β_F) are plotted in Fig. S3.

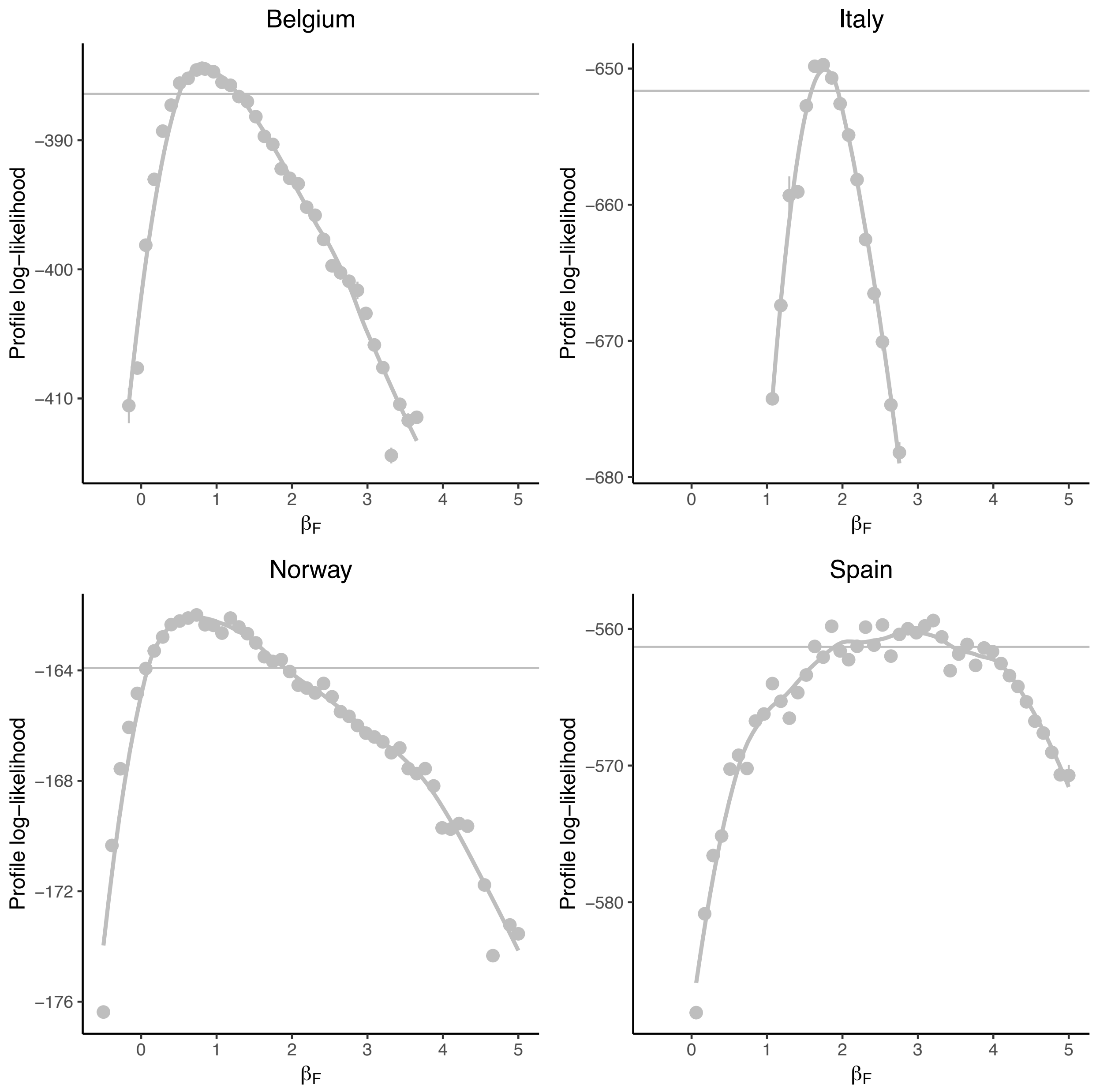

Figure S3: **Log-likelihood profiles for impact of influenza (parameter β_F).** In each panel, the points (line ranges) represent the values of the profile log-likelihood (±SE). The curved grey lines represent the smoothed profiles, calculated using LOESS regression with automatic selection of the span parameter via the function loess.as in the fANCOVA package (span values: 0.52 in Belgium, 0.82 in Italy, 0.38 in Norway and in Spain). The horizontal grey is $0.5 \times \chi^2_{p=0.95,\mathrm{df}=1} = 1.92$ units below the maximum log-likelihood. The y-axis values differ for each panel.

Parameters correlation plot in Spain The correlations between estimated parameters of the base model in Spain are represented in Fig. S4 (see also Table 2). The correlation plots in other countries were qualitatively similar and are not showed here.

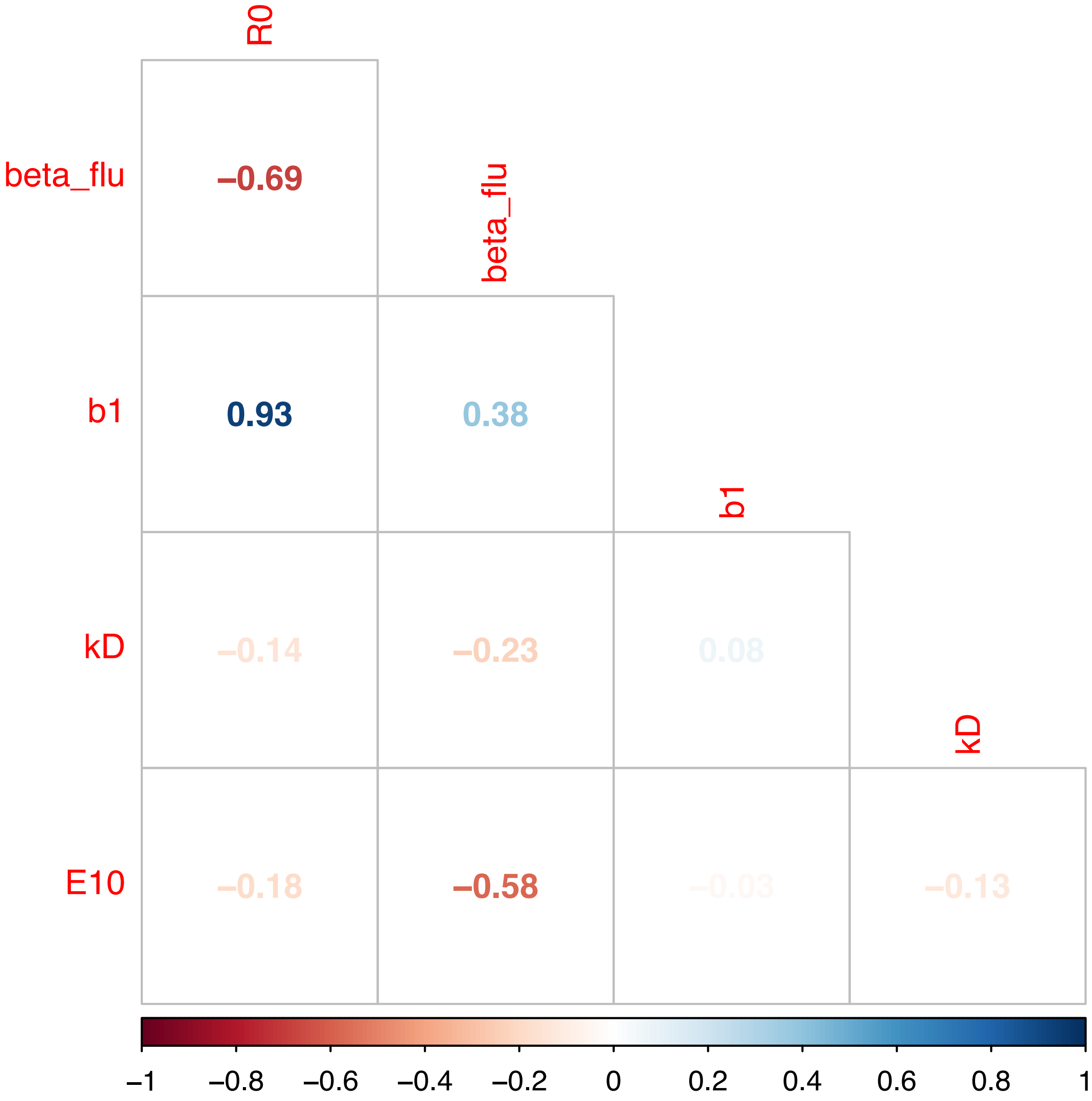

Figure S4: **Parameters correlation plot in Spain.** Represented are the Spearman partial correlation coefficients from 94 MIF runs of the base model in Spain, all within 5 log-likelihood units of the maximum log-likelihood (cf. Table 2).

Model with non-linear function mapping the stringency index to the relative reduction in transmission Although we assumed a simple linear scaling in our base model, it can also be hypothesized that the reduction of SARS-CoV-2 transmission scales non-linearly with the stringency index. For example, super-linear scaling for low values of the stringency index may occur if a potentially high-impact intervention (e.g., lockdown) is implemented early on, such that a modest increase in the stringency index results in

a marked decrease in SARS-CoV-2 transmission. Conversely, sub-linear scaling may also be plausible if potentially low-impact interventions (e.g., border closure) are implemented first. To test those hypotheses, we considered an alternative, non-linear scaling function of the form:

$$r_\beta(t) = f(b_2 \times f^{-1}(\min(1, b \times \frac{si(t)}{100})))$$

where $f(x) = (1+e^{-x})^{-1}$ is the logistic function. Here the extra parameter b_2 controls the slope at the origin, with $b_2 < 1$ representing super-linear scaling at low values of the stringency index, and $b_2 > 1$ super-linear scaling. For $b_2 = 1$, $r_\beta(t) = \min(1, b \times \frac{si(t)}{100})$, such that the base model with linear scaling is nested within this more general model. The corresponding parameter estimates are presented in Table S1, and further discussed in the main text.

Quantity	Belgium	Italy	Norway	Spain
$\log L$ (SE)	–384.4 (0.1)	–649.2 (0.1)	–161.5 (<0.1)	–557.4 (0.2)
$\Delta \log L$ (P)*	0 (1)	0.3 (0.44)	0.3 (0.44)	1.1 (0.14)
R_0	3.1<br>(1.8, 3.4)	1.5<br>(1.1, 1.7)	1.1<br>(1.0, 1.2)	1.3<br>(1.0, 1.9)
b	0.90<br>(0.71, 1.21)	0.67<br>(0.50, 0.90)	0.70<br>(0.65, 0.70)	0.79<br>(0.51, 0.95)
b_2	1.6<br>(0.3, 3.1)	0.6<br>(0.3, 1.2)	3.8<br>NA	0.7<br>(0.3, 1.0)
β_F	0.7<br>(0.7, 2.3)	1.8<br>(1.7, 1.9)	1.0<br>(–0.1, 2.1)	3.0<br>(1.5, 5.3)
k_D	7×10^{-4}<br>$(4, 49)\times10^{-4}$	0.07<br>(0.06, 0.09)	0.15<br>(0.11, 0.47)	0.08<br>(0.06, 0.11)
$E_1(0)$	130<br>(30, 230)	470<br>(160, 970)	540<br>(50, 8200)	350<br>(70, 490)

Table S1: **Point parameter estimates of an extended model with a non-linear scaling function for the stringency index**. *Log-likelihood difference (P-value from a log-likelihood ratio test) with the base model presented in Table 2. The confidence intervals represent approximate 95% multivariate confidence intervals, calculated from the 100 MIF runs as the range of parameters within $\frac{1}{2} \times \chi_{p=0.95,\text{df}=n_\theta}$ units from the maximum log-likelihood ($n_\theta = 6$: number of parameters estimated).

Model with unexplained trend in transmission rate To assess the robustness of our results to potential confounding bias, we considered an extended model that included an exponential trend in the transmission rate:

$$\beta(t) = R_0\gamma(1 - r_\beta(t))\beta_F(t)e^{\tau t}$$

where the trend parameter τ was estimated from the data, in addition to the other parameters. The corresponding parameter estimates are presented in Table S2 and discussed in the main text.

Quantity	Belgium	Italy	Norway	Spain
$\log L$ (SE)	–381.2 (<0.1)	–649.5 (0.3)	–161.4 (<0.1)	–558.1(0.2)
$\Delta \log L$ (P)*	3.2 (0.01)	0.0 (1)	0.4 (0.37)	0.4 (0.37)
R_0	1.2<br>(1.0, 5.0)	1.7<br>(1.1, 4.2)	1.1<br>(1.0, 9.3)	1.1<br>(1.0, 2.0)
b	0.92<br>(0.90, 1.06)	0.60<br>(0.50, 0.79)	0.90<br>(0.50, 1.15)	0.75<br>(0.53, 0.91)
τ ($\times 10^{-3}$ day^{-1})	8<br>(–3, 9)	–3<br>(–8, 2)	8<br>(–18, 9)	3<br>(–4, 6)
β_F	3.6<br>(0.2, 4.4)	1.2<br>(0.5, 2.2)	1.5<br>(–0.5, 3.1)	3.3<br>(1.4, 4.2)
k_D	4×10^{-4}<br>(2, 63)$\times 10^{-4}$	0.08<br>(0.06, 0.10)	0.16<br>(0.11, 0.49)	0.08<br>(0.06, 0.11)
$E_1(0)$	75<br>(10, 130)	440<br>(50, 540)	370<br>(50, 9150)	270<br>(90, 730)

Table S2: **Parameter estimates of an extended model with a trend in transmission**. *Log-likelihood difference (P-value from a log-likelihood ratio test) with the base model presented in Table 2. The confidence intervals represent approximate 95% multivariate confidence intervals, calculated from the 100 MIF runs as the range of parameters within $\frac{1}{2} \times \chi_{p=0.95,\mathrm{df}=n_\theta}$ units from the maximum log-likelihood ($n_\theta = 6$: number of parameters estimated).

Additional sensitivity analyses To verify the robustness of our results, we conducted a number of additional sensitivity analyses. Specifically, we modified the value of 3 fixed model parameters (infection fatality ratio, average onset-to-death time, and average generation time) and we repeated the estimations as before. As shown in Table S3, the estimate of the impact of influenza on SARS-CoV-2 transmission remained consistently above 0 for all scenarios tested. The table also reports the estimates of the model with no impact of influenza on SARS-CoV-2 transmission ($\beta_F = 0$).

**Model**	**Belgium**				**Italy**				**Norway**				**Spain**			
	$\log L$ (SE)	R_0	b_1	β_F	$\log L$ (SE)	R_0	b_1	β_F	$\log L$ (SE)	R_0	b	β_F	$\log L$ (SE)	R_0	b	β_F
Base model	–384.4 (<0.1)	3.4	1.03	0.8	–649.5 (0.1)	1.2	0.53	1.8	–161.8 (<0.1)	2.2	1.05	1.0	–558.5 (0.2)	1.4	0.75	2.4
$\beta_F = 0$	–398.2 (0.1)	5.2	1.09	0	–769.7 (<0.1)	3.4	0.88	0	–164.3 (<0.1)	2.4	1.03	0	–577.4 (0.1)	3.1	1.00	0
$E(T_g) = 5$ days	–385.6 (0.1)	2.7	0.93	0.5	–650.5 (0.1)	1.3	0.50	1.0	–162.3 (<0.1)	1.7	0.87	0.6	–557.6 (0.2)	1.3	0.63	1.5
$E(T_g) = 7.5$ days	–383.5 (<0.1)	3.5	1.06	1.1	–649.5 (0.1)	1.2	0.58	2.3	–161.7 (<0.1)	2.1	1.05	1.3	–558.3 (0.3)	1.2	0.75	4.0
$\mu = 0.005$	–384.6 (0.1)	3.7	1.03	0.6	–649.2 (0.1)	1.3	0.53	1.6	–161.4 (<0.1)	2.1	1.02	0.9	–558.9 (0.1)	1.2	0.66	3.0
$\kappa^{-1} = 13$ days	–392.7 (0.1)	2.1	0.91	3.4	–642.5 (0.1)	1.2	0.50	2.3	–162.7 (<0.1)	1.4	0.80	1.9	–536.8 (0.7)	1.2	0.62	4.5

Table S3: **Sensitivity analyses**.

Model fit to data summary statistics To evaluate the model fit in more detail, we examined the model–data agreement on a number of statistics that summarized important aspects of the mortality data—that is, probes [Wood, 2010, King *et al.* , 2016]). Specifically, we considered the following probes:

- Peak time (in days relative to the start of the study period).
- Peak daily number of deaths.
- Total number of deaths.
- Epidemic growth exponent. According to a previous study [Maier & Brockmann, 2020], we assumed that, until the peak time, the daily number of deaths grew algebraically, i.e., $D(t) \propto t^{\alpha}$. We then estimated the growth exponent α using a log-log linear regression model.

The observed and simulated probe values are plotted in Fig. S5 and discussed in the main text.

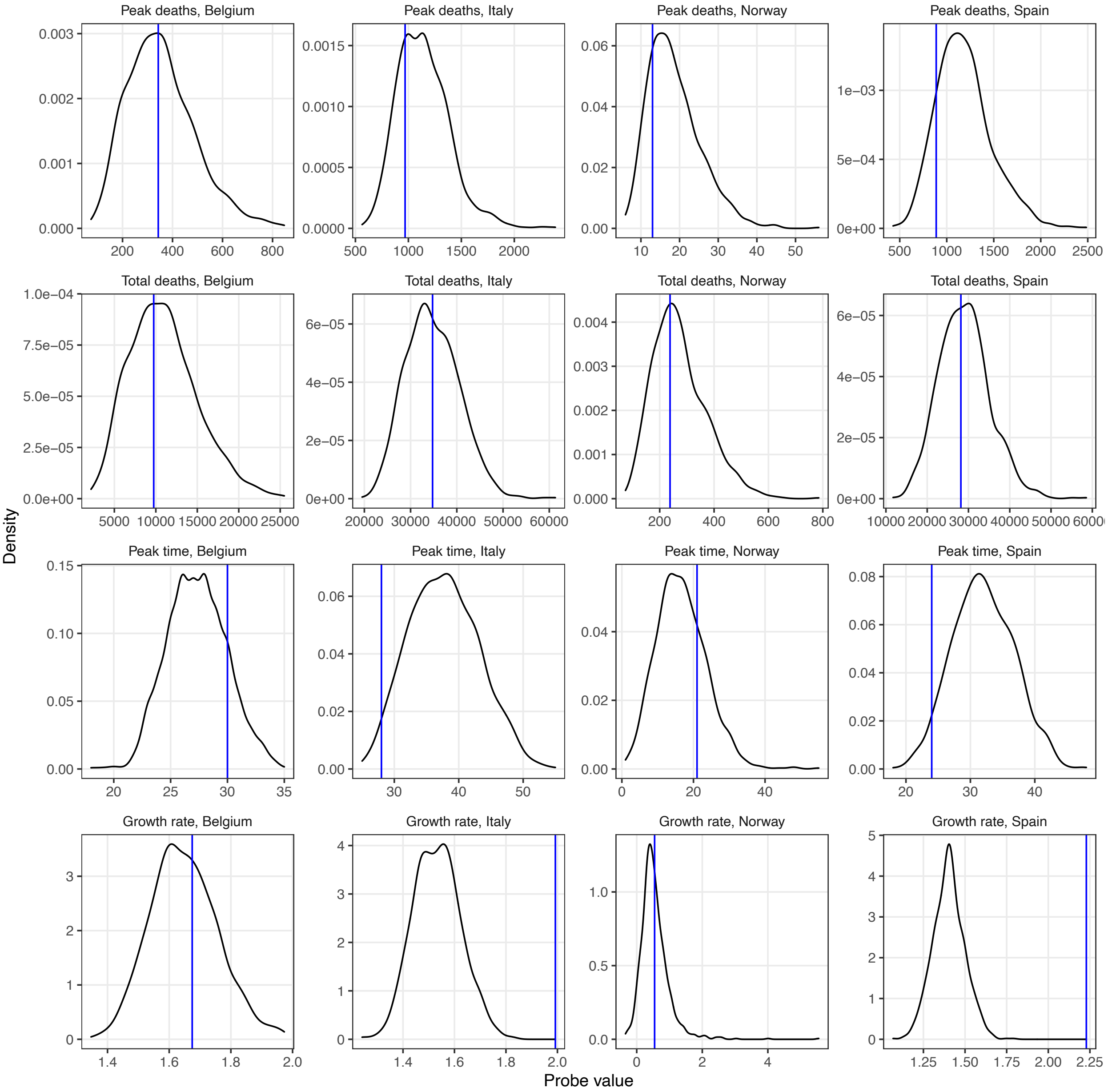

Figure S5: **Model–data comparison on probes**. In each panel, the vertical blue line indicates the observed value of the probe. The x- and y-axis values differ for each panel.

Probability of detecting an influenza–SARS-CoV-2 co-infection To calculate the probability of detecting a co-infection with influenza then SARS-CoV-2, we ran a simulation study. Assuming that influenza infection occurred first, we first generated a sample of influenza incubation periods from a log-Normal distribution with median 1.4 days and dispersion 1.51, based on the results of a previous review [Lessler *et al.* , 2009]. We then generated a sample of detection periods, assuming that influenza could be shed (and therefore detected) up to 4–5 days after symptom onset [Carrat *et al.* , 2008]. Second, we gen-

erated a sample of SARS-CoV-2 infection times, uniformly between the time of infection and the end time of detectability of influenza. Finally, we generated a sample of SARS-CoV-2 incubation periods (from a Gamma distribution with mean 5.7 days [Khalili *et al.* , 2020] and coefficient of variation 0.86 [Flaxman *et al.* , 2020]) and of SARS-CoV-2 detection start times, assuming that SARS-CoV-2 could be detected from 2 to 4 days before symptom onset [Tindale *et al.* , 2020]. In each simulation, we calculated the probability of detecting a co-detection as the fraction of the sample for which the maximal detection time of influenza exceeded the minimal detection time of SARS-CoV-2. The results are presented in Table S4 and discussed in the main text.

**Detection time of influenza after symptom onset**	**Detection time of SARS-CoV-2 before symptom onset**	**Probability of co-detection**
4 days	2 days	0.52
4 days	4 days	0.67
5 days	2 days	0.55
5 days	4 days	0.70

Table S4: **Probability of detecting a co-infection with influenza and SARS-CoV-2**. The results are based on sample size of 10^5; replicate simulations gave identical results, such that the estimates may be considered exact.
